# A protocol for a longitudinal, observational cohort study of infection and exposure to zoonotic and vector-borne diseases across a land-use gradient in Sabah, Malaysian Borneo: a socio-ecological systems approach

**DOI:** 10.12688/wellcomeopenres.17678.1

**Published:** 2022-02-16

**Authors:** Kimberly Fornace, Benny Obrain Manin, Jason Matthiopoulos, Heather M. Ferguson, Chris Drakeley, Kamruddin Ahmed, Koay Teng Khoon, Robert M. Ewers, Sylvia Daim, Tock Hing Chua

**Affiliations:** 1 Institute of Biodiversity, Animal Health and Comparative Medicine, University of Glasgow, Glasgow, UK; 2 Faculty of Infectious and Tropical Diseases, London School of Hygiene & Tropical Medicine, London, UK; 3 Faculty of Medicine and Health Sciences, Universiti Malaysia Sabah, Kota Kinabalu, Malaysia; 4 Sabah State Health Department, Ministry of Health, Malaysia, Kota Kinabalu, Malaysia; 5 Imperial College London, London, UK; 6 East Malaysia Zoonotic and Infectious Diseases Society, Kota Kinabalu, Malaysia

**Keywords:** emerging diseases, socio-ecological systems, coupled human-natural systems, environmental change, landscape epidemiology, cohort study, malaria, arbovirus, zoonosis, vector-borne disease

## Abstract

**Introduction.** Landscape changes disrupt environmental, social and biological systems, altering pathogen spillover and transmission risks. This study aims to quantify the impact of specific land management practices on spillover and transmission rates of zoonotic and vector-borne diseases within Malaysian Borneo. This protocol describes a cohort study with integrated ecological sampling to assess how deforestation and agricultural practices impact pathogen flow from wildlife and vector populations to human infection and detection by health facilities. This will focus on malaria, dengue and emerging arboviruses (Chikungunya and Zika), vector-borne diseases with varying contributions of simian reservoirs within this setting.

**Methods.** A prospective longitudinal observational cohort study will be established in communities residing or working within the vicinity of the Stability of Altered Forest Ecosystems (SAFE) Project, a landscape gradient within Malaysian Borneo encompassing different plantation and forest types. The primary outcome of this study will be transmission intensity of selected zoonotic and vector-borne diseases, as quantified by changes in pathogen-specific antibody levels. Exposure will be measured using paired population-based serological surveys conducted at the beginning and end of the two-year cohort study. Secondary outcomes will include the distribution and infection rates of *Aedes *and *Anopheles *mosquito vectors, human risk behaviours and clinical cases reported to health facilities. Longitudinal data on human behaviour, contact with wildlife and GPS tracking of mobility patterns will be collected throughout the study period. This will be integrated with entomological surveillance to monitor densities and pathogen infection rates of *Aedes *and *Anopheles *mosquitoes relative to land cover. Within surrounding health clinics, continuous health facility surveillance will be used to monitor reported infections and febrile illnesses. Models will be developed to assess spillover and transmission rates relative to specific land management practices and evaluate abilities of surveillance systems to capture these risks.

## Introduction

Zoonotic and vector-borne diseases (ZVBD) are major causes of morbidity and mortality globally. The processes driving ZVBD spillover and transmission risks are determined by interactions between human and natural systems occurring across spatial and temporal scales ^1^. Landscape changes, including land use and land cover changes such as deforestation and agricultural development, alter the socio-ecological systems in which pathogens, disease hosts and insect vectors operate and interact. Such change can either increase or decrease disease risks in different settings, with the potential to undermine control and elimination efforts and lead to outbreaks with devastating human health and economic consequences ^2^. These rapidly changing environments challenge existing disease surveillance systems to detect constantly evolving geographic distributions of infection caused by changing human and environmental systems. New approaches are needed to quantify the resulting pathogen exposure caused by specific land management practices to better target surveillance activities. 

Globally, land use change is one of the major drivers of changes in species distribution and pathogen spillover ^3^. The impacts of these phenomena are increasingly evident in Malaysia; a country with one of the highest rates of landscape change globally with 34% of the land area of Borneo deforested in the past four decades ^4^. Historically, Malaysia has had a highly malaria effective control programme, culminating in the near elimination of nonzoonotic malaria species by 2020 ^5^. However, deforestation has triggered the recent emergence of the zoonotic malaria *Plasmodium knowlesi* in humans ^6, 7^. Since 2004, incidence of reported *P. knowlesi* cases in Malaysia increased over 50-fold, and *P. knowlesi* is now the main cause of human malaria, with cases highly concentrated in Malaysian Borneo ^8, 9^. Malaysia is also experiencing exponential increases in mosquito-borne arboviruses, with landscape change hypothesised as the main driver of recent expansion ^10^. For example, while dengue is commonly associated with increased urbanisation, the incidence of dengue in rural areas of Malaysia is now approaching levels observed in urban centres ^11, 12^. This may be attributed to the importance of sylvatic dengue reservoirs in Malaysia. For example, pilot studies identified high rates of dengue seropositivity in macaques (100%, 9/9) and *Aedes* mosquitoes in rural areas, suggesting transmission may be partially driven by zoonotic spillover (Fornace *et. al*, unpublished; ^13^). Similarly, both chikungunya and Zika viruses have been isolated in non-human primates in Borneo and sporadic outbreaks are reported in rural populations in Malaysia ^14– 16^. This suggests landscape changes could alter the relative importance of sylvatic transmission with potential to generate contradictory effects on human risks. For example, development can lead to higher rates of human-to-human transmission but reduced risk from zoonotic spillover. Together, these data indicate landscape changes are substantially altering ZVBD epidemiology within this region and highlight the need to understand how specific land management practices impact infectious disease risks.

Despite extensive research identifying environmental risk factors and landscape change impacts on vector populations (e.g. 17– 19), ZVBD risks resulting from specific land use patterns are rarely quantified and, as a result, ZVBD risks rarely inform land planning decisions. Developing actionable predictions of disease risks is challenging due to the complex, nonlinear relationships characterising landscape change effects on coupled human-environment systems ^20^. Coupled human-environment systems, also called socio-ecological systems, refer to systems with feedbacks between environmental and societal dimensions. For example, while human activities shape landscapes, these environments in turn influence human mobility, behaviour and economic status ^17, 21^. Assessing impacts of these environmental changes requires integrating data on human behaviour, ecology and pathogen transmission. This may be further challenged by the limited detection of infections within human populations due to variations in health-seeking behaviour, misdiagnosis and frequent asymptomatic infections. Serological surveillance provides new opportunities to measure previously undetected exposure to multiple pathogens simultaneously ^22^. By measuring long-lived antibody responses, these methods substantially increase the probability of detecting rare events and can be used to estimate transmission intensity over time or across different ecological types ^7, 23, 24^. Integrating serological data with detailed field studies can lead to new insights on how land management impacts transmission.

**Figure 1.**
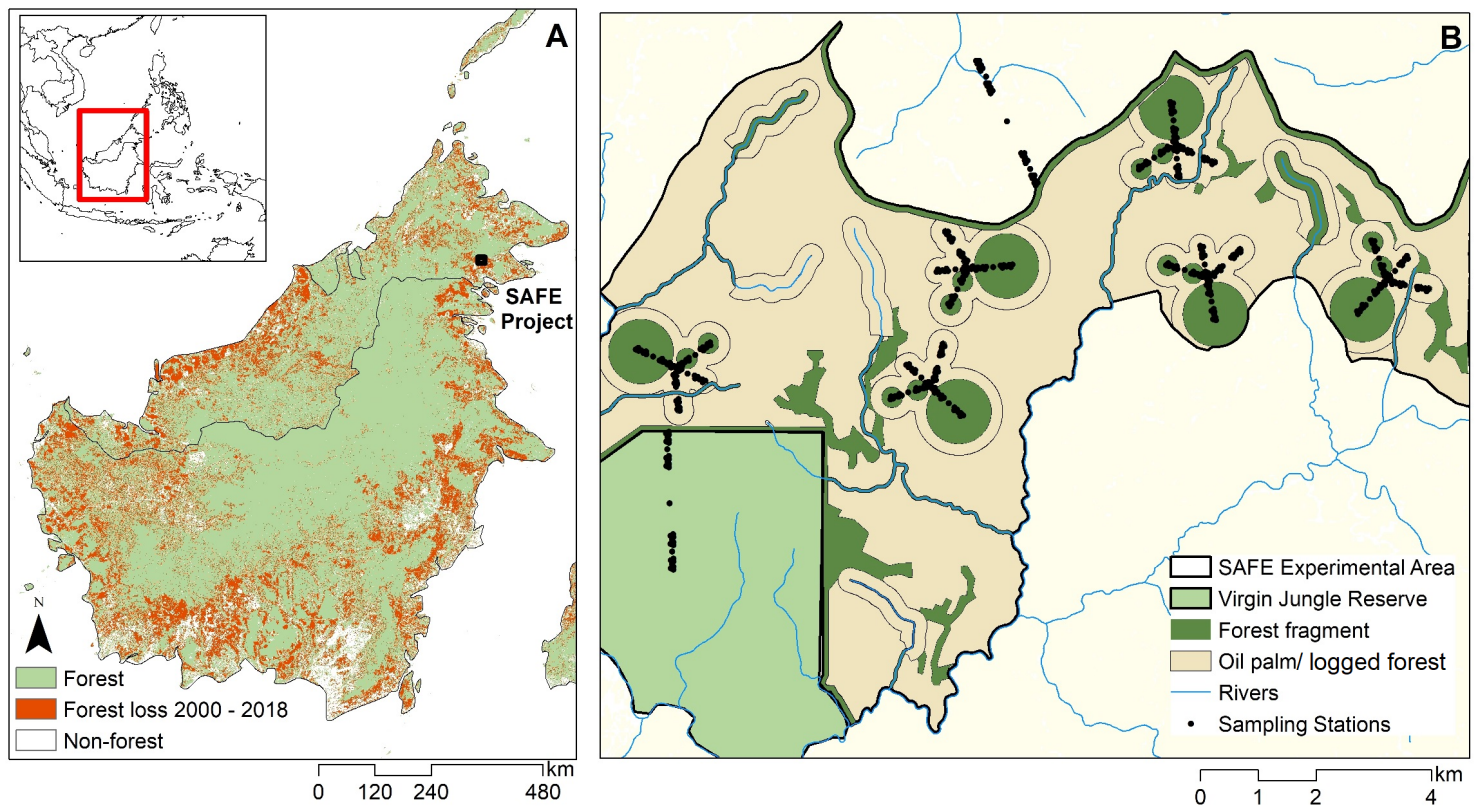
**A.** Forest cover change in Malaysian Borneo and the Southern Philippines, **B.** SAFE Project experimental forest modification site.

This study will investigate the effects of land management on ZVBD risks within large-scale experimental land use study, the Stability of Altered Forest Ecosystems (SAFE) project in Malaysian Borneo ( SAFE Project) The SAFE project uses an experimentally fragmented landscape (100km ^2^) and sampling within conservation areas (1020km ^2^) and oil palm plantations (30km ^2^) to monitor the effects of tropical forest modification on biodiversity and ecological processes across varying spatial and temporal scales ( Figure 1 ^25^). This study will build on this experimental ecosystem to examine how specific forest management practices and agricultural developments influence ZVBD spillover and transmission risks.

### Aims and objectives

The overall aim of this study is to assess how land use impacts human exposure to malaria, dengue and arboviruses (Chikungunya and Zika viruses) in Malaysian Borneo. This will quantify how specific land management patterns impact socio-ecological systems to drive pathogen spillover and transmission risks. The primary outcome of this study will be the change in magnitude in antibody responses in individuals working and residing within forests, plantations, farms and rural villages surrounding the SAFE Project. Exposure will be measured by the presence of antibodies to pathogen-specific antigens. As secondary objectives, this study will collect data on distribution and infection levels of mosquito vectors, assess human behaviours and measure the incidence of malaria, dengue and arbovirus infections from clinically reported cases. Specific objectives include:

1) To determine the incidence and serological exposure to malaria, dengue and selected arboviruses in human populations residing and working within in the region surrounding the SAFE Project;
2) To characterise human behaviour and movement patterns influencing pathogen exposure risks;
3) To describe the spatiotemporal distribution of *Anopheles* and *Aedes* mosquito vectors relative to land type and wildlife reservoir abundance; and
4) To estimate human exposure to malaria, dengue and arboviruses associated with specific land management practices.

## Protocol

### Study design

**Figure 2.**
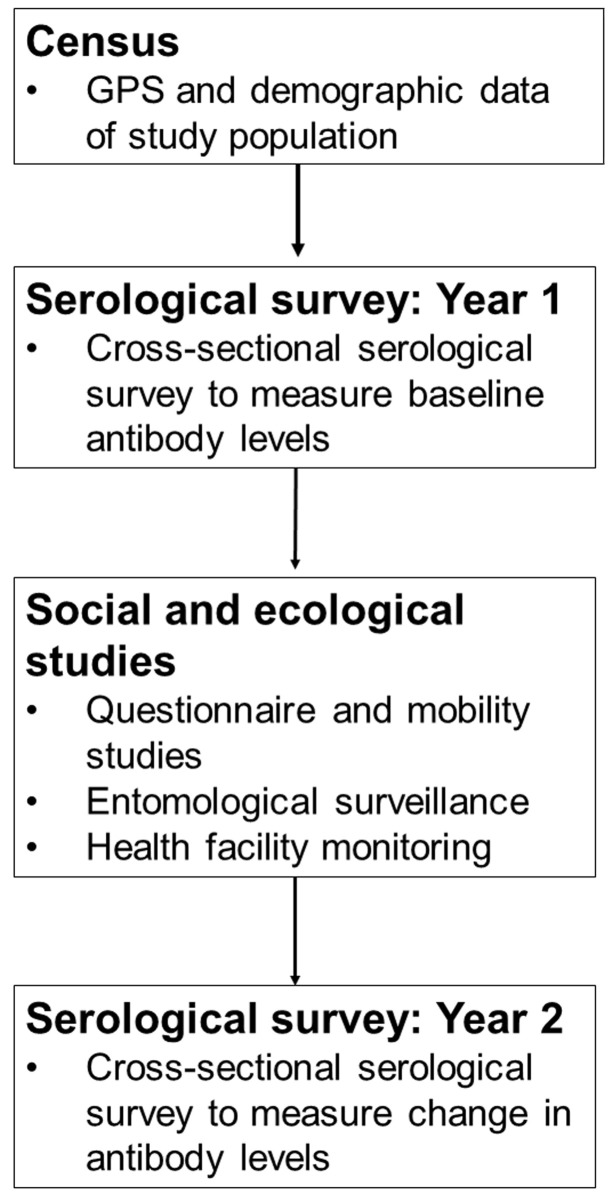
Workflow of study activities.

This study will be a prospective longitudinal cohort study to evaluate exposure to malaria, dengue and other arbovirus infections in human populations living and working in the vicinity of the SAFE Project in Tawau District, Sabah, Malaysia. Exposure to selected ZVBDs will be assessed using paired cross-sectional serological surveys at the start and end of the two-year study period. During this period, continuous health facility surveillance will record reported febrile illnesses and household locations. Throughout the study, randomly selected individuals residing in the study area will be asked to participate in questionnaire surveys and GPS tracking studies. Longitudinal entomological sampling will be conducted to evaluate the density of *Anopheles* and *Aedes* mosquito vectors relative to habitat. The full study design is outlined in Figure 2.

### Study site

This study will be conducted in populations residing near the SAFE Project in Tawau District in Malaysian Borneo ( Figure 1A). The SAFE Project site encompasses a gradient of habitat modification including old growth rainforest, logged rainforest, logged and fragmented rainforest and intensive agriculture (oil palm). These habitat modification levels additionally incorporate different land use and disturbance intensities, such as different logging frequencies within forests and various plantation ages and canopy cover levels. Fragmented forest sites include six replicate blocks containing a total of 42 experimental circular forest fragments of 1, 10 or 100 hectares and embedded in a matrix of heavily damaged, salvage-logged forest ( Figure 1B). Across these sites, a fractal sampling design was established to collect biodiversity and ecological data across spatial scales ^25^. This includes a network of camera traps and extensive data on *Aedes* and *Anopheles* mosquito ecology (e.g. 19, 26, 27).

Within the area surrounding the SAFE Project, the human population is rural and includes forestry and plantation camps, villages and a larger town with multiple small government clinics and one district hospital. All target ZVBD have been reported in this region, including increasing numbers of human *P. knowlesi* cases since 2015 ^8^. As with all of Malaysia, no indigenous non-zoonotic malaria cases have been reported since 2018 ^5^. All target diseases are notifiable and there are active vector-borne disease control and elimination programmes within this region. The climate is equatorial and tropical with monthly variations but no clear seasonal patterns in rainfall or vector-borne disease reports ^28^.

### Study participants

The study population will include communities within the vicinity (less than 50km) of the SAFE Project in Tawau, Sabah, Malaysia. To define this population, a complete census will be conducted to record basic demographic data and household GPS coordinates for all individuals residing within this area. Throughout the study period, this census will be updated monthly to reflect individuals moving into or out of this area.

### Inclusion criteria

Individuals will be eligible for this study if they reside within the defined study area. Residence is defined as spending more than 30 continuous days within the study area prior to the survey activity. For example, individuals moving into the study area will be eligible for inclusion into GPS tracking or serological survey activities once they have resided within the study site for 30 days.

### Exclusion criteria

Individuals will be excluded from this study if they do not reside in the area, do not consent or are under the age of 3 months. For GPS tracking studies, individuals will be excluded if they are under the age of 8 years old.

### Withdrawal criteria

Subjects can choose to withdraw at any time. If the subject chooses to withdraw from the study after previously providing consent, their specimens will be destroyed and data will not be used for analysis unless permission is explicitly given. A subject can withdraw consent at any time by contacting the study team (see ethical considerations).

### Sample size

Sample sizes have been calculated to detect a change in the magnitude of antibody responses from two paired samples collected at the beginning and the end of this study. Based on previous estimates of 5% annual seroconversion rates (SCR) to *P. knowlesi*, 3% to dengue within rural Sabah, Malaysia, expected low incidence of other arbovirus infections ( Table 1) and 5% probability of type-1 errors, McNemar’s test estimated 645 paired samples could identify a 2.5% annual increase in seropositivity with >95% power ^7, 16, 29^. As relatively high loss to follow up is expected during the study period due to population movement, the sample size for serological surveys will be 870, including a 20% loss to follow up and 15% non-response rate.

**Table 1.** Previously reported annual seroconversion rates (SCR) for selected ZVBDs.

Pathogen	Site	SCR or Incidence
*P. knowlesi*	Northern Sabah, Malaysia ^7^	0.051 (0.048-0.054) serological exposure within past year
P. falciparum *		SCR: 0.006 (0.005-0.006)
P. vivax *		SCR: 0.004 (0.008-0.022)
Dengue		SCR: 0.028 (0.019-0.042)
Chikungunya	Cebu, Philippines ^29^	3.1 (3.0-4.0) outbreaks/year; 0.22 seroprevalence
Zika	Malaysia ^16^ Philippines ^30^	Unknown, sporadic cases, historical transmission

* Nonzoonotic species no longer reported in Malaysia but indicative of malaria transmission rates

### Study procedures

**Figure 3.**
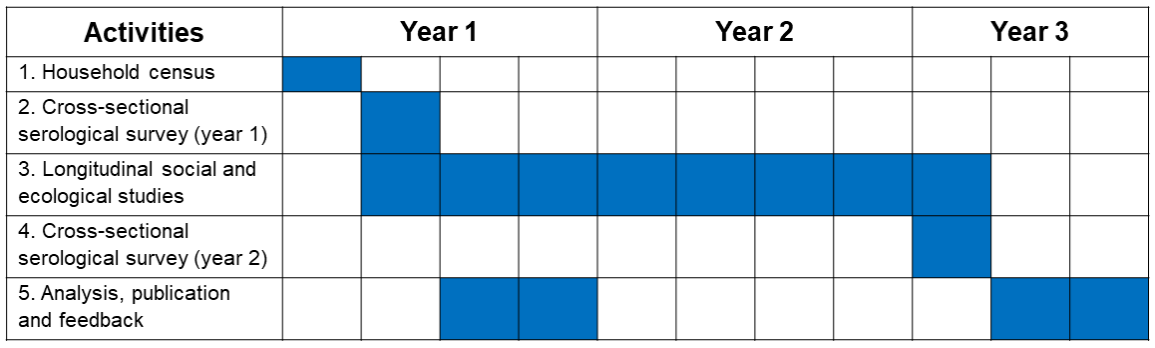
Timeline for study activities.

This study will consist of a two-year cohort study with parallel ecological monitoring of environmental variables and mosquito densities. The study activities and proposed timelines are outlined in Figure 3.

Prior to commencing any activities, meetings will be held with community leaders and members to introduce the study and answer any questions. Additional meetings and community sensitisation activities will be conducted with the SAFE Project staff and key stakeholders, such as plantation and forestry workers. Based on these meetings, a study area will be defined with a complete list of kampungs (villages). Kampung lists will include villages as well as plantation and forestry camps. Following initial meetings, all households within the study areas will be enumerated and assigned a unique identification code linked to the household GPS coordinates. If no household members are present at the time of enumeration, fieldworkers will consult with the head of the village and may revisit the household or attempt to contact residents by phone.

Results from this census will be employed to identify the sample population using a two-stage sampling design. At the first stage, kampungs (the primary sampling unit) will be selected from updated kampung lists obtained from the census. Kampung selection will be stratified based on indicators of land cover into three strata: forest areas, plantation camps and residential villages. At the second stage, a fixed number of households will be randomly selected from each kampung using a random selection function in R statistical software to select household prior to survey activities. All individuals above the age of 3 months in these selected households will be asked to participate in the cohort study, with a total sample size of 870 individuals (approximately 220 households). For children under 18, permission will be requested from parents or guardians (see ethical considerations). Two cross-sectional serological surveys will be conducted at the start and end of the cohort study.

Selected households will be visited and all individuals who have resided in the household for the previous month will be asked to participate in the serological surveys. The study will be explained by a trained research fieldworker and written informed consent will be obtained during a household visit. Consenting individuals will be interviewed on demographic characteristics, movement patterns, travel history, health seeking behaviour and land use practices. Electronic forms of questionnaires will be designed using Open Data Kit or similar software and data will be collected using encrypted electronic tablets. A combination of village meetings and call-backs will be used to trace non-responders.

Samples will only be collected during the two serological surveys. A trained fieldworker will perform finger prick blood sampling to collect a maximum of 500μl of whole blood using an EDTA micro-container and three blood spots (20μl each) on filter paper ^31^. All blood samples will be processed and stored for serological analysis of exposure to malaria, dengue and arboviruses. EDTA blood samples will be centrifuged and plasma separated for serological assays with the cellular fraction retained for molecular analysis of infection. Blood smears for malaria parasites or malaria rapid diagnostic tests (RDTs) will also be prepared for all consenting individuals. Febrile individuals or individuals with a history of fever within the past 24 hours will have results assessed within 48 hours to ensure any infected individual receives appropriate treatment. Subjects with positive tests will be referred to the local district referral hospital (Tawau District Hospital) where they have free access to treatment and management as per standard Malaysian Ministry of Health guidelines. All other participants will be provided with their study code and contact information for the study team if they have any questions about their results.

Sero-epidemiological methods will be used to detect human antibodies to pathogen-specific antigens. These antibodies reflect exposure to previous infection and can be used to characterise disease transmission (e.g. 22, 24, 32, 33). This will use a custom-developed antigen panel on a multiplex bead assay to measure the magnitude of short- and long-term antibody responses to specific pathogens ^7, 34^. Plasma will also be assayed to assess exposure to other infectious diseases of interest to the Malaysian Ministry of Health. Assays will be conducted in Malaysia with a subset of samples run for quality control at the London School of Hygiene and Tropical Medicine in London, UK. Specimens will be stored at -20°C, following human tissue storage procedures established by the Universiti Malaysia Sabah.

Throughout the two-year study, rolling cross-sectional surveys of individuals residing within selected kampungs will be conducted to characterise movement patterns and disease prevention practices. Consenting, randomly selected individuals will be interviewed on occupational activities, mobility, travel history and health behaviours. Individuals aged 8 years and over will be asked to participate in GPS tracking studies. These individuals will be selected from the communities included within serological surveys, with final sample sizes to be confirmed based on demographic distribution of study participants. During these studies, participants will be given a GPS tracking device (e.g. QStarz BT-Q1000XT or a mobile phone) and asked to wear the GPS device at all times outside of the house, including on trips overnight. The device will be programmed to record a GPS location every minute for a minimum sampling period of two weeks, with participants informed about GPS tracking data, interviewed about the use of GPS tracking devices and data downloaded and manually checked by study personnel ^17^. During this period, a research assistant will regularly visit to check the device and change batteries. These data will be used to develop predictive spatiotemporal maps of human space use relative to habitat.

Additionally, to assess health seeking behaviour throughout this two-year period, continuous health facility monitoring will be established at health facilities serving the study area. This will capture anonymised data on the numbers of febrile patients attending the clinic daily, number of diagnosed infections and the residence locations of these cases. These data are routinely captured as part of Malaysia’s vector-borne disease surveillance systems. Additionally, consenting health facility attendees will be asked to provide more detailed information on their household location and movement patterns ^35^. These data will be collected at clinics by health facility staff using encrypted tablets with offline satellite maps to report and geolocate the residences and workplaces of health facility attendees seeking treatment for febrile illnesses ^36^. Other explanatory variables, including demographic information and occupational activities, will be collected using online questionnaires. Data will be integrated into models of infection rates and used to assess the sensitivity of passive surveillance systems.

Ecological studies of the surrounding area will be conducted in parallel to cohort activities. Detailed data on land cover, topography and the distribution of wildlife populations will be obtained from the SAFE Project. Throughout the study period, longitudinal entomological sampling will be conducted to evaluate the density of *Anopheles* and *Aedes* mosquito vectors across the study site. The primary outcome of the entomological surveillance will be the abundance of host-seeking vectors, which will be used to develop predictive spatiotemporal models of vector density. Additionally, mosquitoes will be assayed for pathogen presence with molecular methods. Locally validated methods of trapping host-seeking vectors using a Mosquito Magnet trap with an attractant, carbon dioxide and counterflow technology to attract and capture mosquitoes (Mosquito Magnet; Woodstream Corporation Inc., Lancaster, USA) will be used to collect *Aedes* and *Anopheles* vectors; this method has shown strong correlation with human landing catches in this region ^37, 38^. For *Aedes* species, sticky traps will be used to increase the probability of detecting infected mosquitoes ^39^. No human landing catches will be used to collect mosquitoes due to ethical considerations.

Entomological sampling locations will be positioned across the study site to capture the spatial distribution of mosquito abundance. The study site encompasses plantation and village areas as well as old growth and degraded forests with varying levels of fragmentation ^25^. Entomological sampling will be conducted at eight separate locations for two days/nights at monthly intervals throughout the study. To determine the locations of entomological sampling sites, we will use a simulation-based approach designed to optimise habitat fragmentation studies ^40^. This approach extends commonly used geostatistical survey designs, in which survey locations are positioned to maximise predictive abilities and minimise survey effort by leveraging spatial autocorrelation between survey points ^41, 42^. Geostatistical sampling designs will be adapted to maximise habitat differences between sampling locations by integrating metrics on land cover, fragment size and distance from forest edges ^40^. Previously collected mosquito sampling data from the SAFE Project (e.g. 19, 26) will be used to refine and validate simulations.

### Statistical analysis plan

For the primary outcome, paired serological data on the magnitude of antibody responses will be used to calculate pathogen-specific forces of infection within the study area ^43^. Models classifying serological exposure will be informed by extensive training datasets on serological responses of exposed and unexposed populations within this region (e.g. 7, 34). These will use Bayesian latent modelling approaches to estimate the (unobserved) true incidence of infections from imperfectly detected serological measurements and reported incidence of infections at health facilities. Additionally, risk factor analysis will be used to identify key demographic, behavioural and spatial risk factors for individual-level seropositivity against specific pathogens within this population, using random effects to adjust for clustering within households and villages.

**Figure 4.**
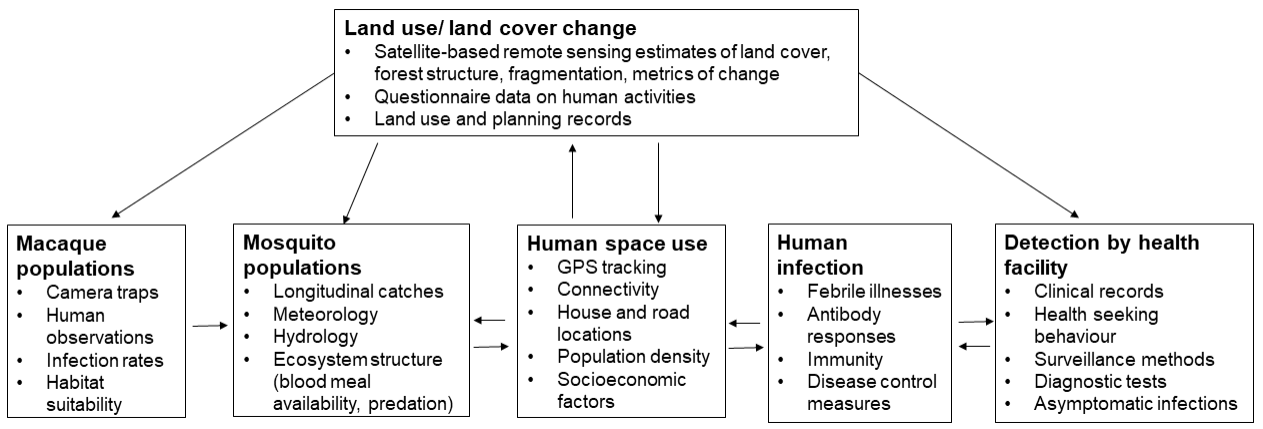
Key data inputs into modelling pathogen flow from wildlife through to detection at health facilities.

To assess the role of land management practices on spillover and transmission, separate spatially and temporally explicit component models will be developed for human and ecological factors impacting pathogen risks. Empirical data will be used to develop predictive spatiotemporal models for human space use (measured as time per grid cell location from GPS tracking data), healthcare coverage (probability of attending health facilities within a given time) and *Anopheles* and *Aedes* vector abundance (from entomological catch data). Models will explore methods of accounting for observation biases and spatial and temporal autocorrelation when relevant ( Figure 4). Models will be assessed through posterior predictive checks and fit with test and training datasets, validating with independent data when possible. For all predictive models, remote sensing derived environmental covariates, including land cover and meteorological factors, will be assessed for inclusion as explanatory factors. Each component model will be fit separately first before exploring modelling approaches to estimate pathogen flow through different populations relative to land management practices ^44^.

All data analysis will be performed using R statistical software ^45^. Hierarchical Bayesian modelling approaches will be implemented in open-source software such as Integrated Nested Laplace Approximation (INLA) using R statistical programming language ^46^.

### Ethical considerations and permissions

This study will be conducted in compliance with ethical principles outlined in the Declaration of Helsinki and Malaysian Good Clinical Practice Guidelines. All procedures will require approval from the Malaysian National Ethics Committees and relevant Institutional Review Boards. Additionally, permission will be required from the Sabah Biodiversity Council to conduct entomological sampling and access environmental data. Access to the SAFE Project site will require protocol review and permission from the SAFE research network through an established review process.

For human studies, written informed consent or assent will be obtained from all study participants. The English versions of information sheets and consent forms will be translated to Bahasa Malaysia and the back translations will be submitted to the Malaysian Research Ethics Committee. Written informed consent will be obtained from all eligible adult participants and the parents or guardians of child participants (< 18 years old) prior to the collection of information. In order to explain the study, the information and consent forms will be read out to the participant or the participant’s guardian in Bahasa Malaysia or English and they will be invited to ask questions. If they agree, the participant or the guardian will sign the consent form. If the participant is illiterate, thumbprints will be used for the consent form. Assent will also be gained on a separate form for appropriate adolescents under the age of 18 using the same signature and thumbprint methods. If the potential participant does not give consent or does not meet the study inclusion criteria then the reason for non-participation will be documented in the recruitment log. Separate consent will be obtained for serological sampling for the cohort study and GPS tracking activities.

The risks associated with participating in this study are minimal. Serological surveys will involve finger prick blood sampling (<500μl) taken from all consenting participants. While mild discomfort may be felt during and after the finger prick, this presents minimal risks to participants. Blood will be collected by trained personnel to ensure safety. All participants will benefit from knowing whether they have malaria infections. Participants with positive tests will be referred to local hospital clinics where they will be treated free of charge. Additionally, this study will provide a better understanding of the diseases being assessed to inform future surveillance and control activities.

### Data management and confidentiality

A primary ethical consideration for this study is maintaining data privacy and confidentiality. Study codes will be used to identify all participants and households. Electronic forms of questionnaires will be designed using ODK or a similar software program. Data will be collected using encrypted tablets and transferred to a central data repository. Data will be password protected and encrypted and all information on participants will remain confidential. Secured network file systems, which will be institutionally maintained with regular automated backups, will be utilised to store electronic data. All data involving human participants will be stored on secure servers accessible only to study personnel. Data will be collated after securing informed consent and destroyed if consent is withdrawn. Data will be anonymised as much as possible, coding and removing personal identifiers. GPS locations of households and GPS tracking data from individuals will be treated as identifiable information and access will be limited to designated study personnel who will be required to maintain confidentiality. No personal information will be shared with third parties and any maps or spatial data published will be modified so it cannot be linked to participants.

All electronic and paper data access will be restricted to authorised study personnel only. Data, including laboratory notebooks, will be archived according to Universiti Malaysia Sabah policies and kept for a minimum of ten years. All electronic data, including spatial data, will be stored on secured servers for a minimum of ten years. Data collection, documentation, storage and preservation will follow the University of Glasgow Good Management of Research Data Policy and will be stored on systems compliant with the ISO27001 Information Security management standards. Details of blood samples will be entered into existing computerised databases on supported network file systems complying with the UK Human Tissue Act.

## Dissemination

All study results will be published in open access journals and presented to the Ministry of Health and other relevant organisations in Sabah, Malaysia. Summaries of research findings will be presented to local stakeholders and communities involved within this study. While ethical considerations preclude sharing identifiable data on human populations, other datasets (e.g. mosquito data) will be shared through publicly available research data repositories maintained by the University of Glasgow and SAFE Project. All statistical code developed during this project will be shared through GitHub repositories and published as supplements to publications to ensure reproducibility. Research outputs and tools will also be shared to wider audiences through networks such as the Health GeoLab Collaborative and Asia Pacific Malaria Elimination Network.

## Conclusions

This study will be the first to describe disease transmission and exposure within a large-scale experimental landscape. This will examine how specific land management practices impact zoonotic and vector-borne disease dynamics, developing methods for characterising health impacts of environmental changes in socio-ecological systems. This study should provide new insights into mechanisms driving disease emergence in changing landscapes and identify future surveillance and control priorities.

## Study status

This study is currently under review with relevant ethics committees, with study activities planned to commence in September 2022, conditional on COVID restrictions.

## Data availability

### Underlying data

No underlying data are associated with this article.

### Extended data

Zenodo: SAFE Sampling area borders. https://doi.org/10.5281/zenodo.3697804 ^47^

Data are available under the terms of the Creative Commons Attribution 4.0 International license (CC-BY 4.0).
