# Supplementary material for: A protocol for a longitudinal, observational cohort study of infection and exposure to zoonotic and vector-borne diseases across a land-use gradient in Sabah, Malaysian Borneo: a socio-ecological systems approach
Source: Wellcome Open Res. 2022 Feb 16;7:63. [Version 1] doi: 10.12688/wellcomeopenres.17678.1 (PMC8886174; doi:10.12688/wellcomeopenres.17678.1)
Supplement: Peer review history

# Peer review: Reviewer response for version 1

**DOI:** 10.21956/wellcomeopenres.19556.r48777

**Published:** 2022-02-28

**Authors:** Gabriel Zorello Laporta

**Affiliations:** 1 Centro Universitário Faculdade de Medicina do ABC (FMABC), Santo André, Brazil

In plain text, authors will test 645 human subjects in given period and then two years later to obtain health status variation as 0, no change; 1, change to risk; -1, change to protection per zoonotic and vector-borne disease agent (Plasmodium, DENV, CHIKV, and ZIKV). They will adjust regression model having status variation as the response variable in function of explanatory variables: (1) human space use; (2) healthcare coverage; (3) mosquito vector abundance; (4) environmental covariates. It’s therefore a strong study design.

 My only concern is with authors stating infections in *Aedes *and *Anopheles *will be tested in the Abstract; however, this method (rather complex) is not contained in this protocol of study.

Is the study design appropriate for the research question?

Yes

Is the rationale for, and objectives of, the study clearly described?

Yes

Are sufficient details of the methods provided to allow replication by others?

Yes

Are the datasets clearly presented in a useable and accessible format?

Not applicable

Reviewer Expertise:

Disease Vector Ecology

I confirm that I have read this submission and believe that I have an appropriate level of expertise to confirm that it is of an acceptable scientific standard, however I have significant reservations, as outlined above.

# Peer review: Reviewer response for version 1

**DOI:** 10.21956/wellcomeopenres.19556.r48776

**Published:** 2022-02-23

**Authors:** Jessica E. Manning

**Affiliations:** 1 Laboratory of Malaria and Vector Research, National Institute of Allergy and Infectious Diseases, National Institutes of Health, Bethesda, MD, USA

This is a well-written and intriguing protocol of public health relevance today. The unique opportunity afforded by an experimental landscape is a major benefit and the authors created a comprehensive, multi-pronged approach to analysis. This protocol submission describes a longitudinal observation cohort over two years that will incorporate a serosurvey in ~870 people for NVBD pathogens at beginning and end (plus demographic and risk factor data), monthly entomological collections, subset of mobility studies for both humans and macaques, and collection of population-level health records recording case counts of febrile illness, etc.

 This is well-thought out and I have minor comments on study design that I believe are valid for following antibody responses to arboviruses. The authors plan to sample at the beginning of Year 1 and the end of Year 2 (a 24-month time frame) and they estimate a seroconversion rate of 2.5% annual increase. Given the increasing dengue burden in Sabah as laid out by Murphy *et al.,* 2020, it highly likely that most of the population has developed multitype immunity. Meaning that of the 870 persons sampled, unless they are all children under age 10, they will likely not be arboviral-naïve and it will make it much more difficult to follow their antibody responses and the study would be drastically underpowered for arboviruses. However, this would depend to some extent on the planned assay but I cannot tell from the draft what multiplex bead array will be used as the references are all for *Plasmodium* infection - not arboviruses. However, with only 500ul of whole blood collected - this won't render enough sera to do serotype-specific neutralization assays that would allow for quantitative titers. I presume the serological analysis is a pan-dengue assay (not serotype-specific) for IgM or IgG. This approach may work for Zika or Chik since most of the population may be naïve, but I think you will be hard pressed to calculate an FOI from serological data from 870 people, the vast majority may be immune to at least one serotype. I agree this work with your naïve population. My questions are:

- What percent of your enrolled population will be naïve?
- Is the bead-based assay serotype specific for IgG and IgM and how do you account for the cross-reactivity from multiple endemic flaviviruses?
- How will JEV vaccination status or timing of Sabah impact either the baseline survey or following up magnitude of change in antibodies?
- If you could detect a serotype-agnostic change in antibody magnitude in a person who initially dengue-monotypic with your assay, is 24 months too large of time window and antibodies from a heterotypic infection will have decreased back to resting state? 

My only other comment is that two days of mosquito trapping per month for two years at eight sites may result in a very low number of infected *Aedes* mosquitos. We find it very rare to actually find infected pools even when collecting in the midst of dengue epidemics (even in hospitals with viremic patients everywhere being bitten). I would temper any expectations that this will yield much data in terms of infection, but may certainly help with abundance measures although we know that this can be wildly inconsistent and variable (but even better - can you assess host-vector contact via serology).

 The mobility studies and ecological studies are fascinating, and I certainly don't have the expertise to comment on them. The continuous health facility monitoring - we have challenging making more granular conclusions from these data sources - but I agree it is important to capture.

 Overall, I believe this is an excellent protocol with a few details to be provided regarding arboviral Sero-epidemiology.

Is the study design appropriate for the research question?

Partly

Is the rationale for, and objectives of, the study clearly described?

Yes

Are sufficient details of the methods provided to allow replication by others?

Yes

Are the datasets clearly presented in a useable and accessible format?

Yes

Reviewer Expertise:

Dengue, Aedes, mosquito saliva, Sero-epidemiology, metagenomics

I confirm that I have read this submission and believe that I have an appropriate level of expertise to confirm that it is of an acceptable scientific standard, however I have significant reservations, as outlined above.

# Peer review: Reviewer response for version 1

**DOI:** 10.21956/wellcomeopenres.19556.r48775

**Published:** 2022-02-22

**Authors:** Konstans Wells

**Affiliations:** 1 Department of Biosciences, Swansea University, Swansea, UK

This protocol presented by Dr Kimberly Fornace and an interdisciplinary and highly experienced team describes a planned observational cohort study to explore possible exposure to selected zoonotic pathogens and changes the antibody response over a one year of a human population residing in a tropical forest production landscape in Malaysian Borneo. Health facility monitoring data will be used to report febrile illness incidences. In addition, mosquito vectors will be collected in the field in order to describe spatiotemporal changes in their occurrence and infection status.

 The study is proposed to investigate the human population near the Stability of Altered Forest Ecosystems project area (SAFE: https://www.safeproject.net), a study area of ~7,200 ha of forest fragments and a Virgin Jungle Reserve logged forest of Ulu Segama Forest Reserve.

 This is a very timely project and hopefully this will be a great source of insights of how of the socio-ecological conditions may affect vector-borne disease spread in tropical forest landscapes and how exposure risk can be minimized.

 Considerable thought has been put into this protocol and most sampling procedures have been described in sufficient detail to provide an informative overview of this study. However, I found the description of the study area slightly confusing, given that the area of the SAFE project differs from the much larger area from which relevant data of the residential human population and health clinics can be sampled.

 To allow a broader readership to benefit from this protocol, I would welcome more detailed information on the expected spatial extent of the study area from which the various data types will be collected and also more details of how various data types will be matched.

 Some clarifications might include:

- A revision of Figure 1B to show the location of major focal villages and health clinics and better put the 7,200 ha of the SAFE project site in context of a study area of ~50km around this site.
- Details of the planned sampling design for mosquitos (the current version states that sampling will be conducted across the study site, which is a large undertaking).
- Details on the data origin and sampling design for camera trap and “macaque populations” data as mentioned in Figure 4.

Is the study design appropriate for the research question?

Partly

Is the rationale for, and objectives of, the study clearly described?

Yes

Are sufficient details of the methods provided to allow replication by others?

Yes

Are the datasets clearly presented in a useable and accessible format?

Not applicable

Reviewer Expertise:

Biodiversity, disease ecology, Borneo

I confirm that I have read this submission and believe that I have an appropriate level of expertise to confirm that it is of an acceptable scientific standard.
